# Combating and overcoming drug resistance in cancer

**DOI:** 10.3389/fphar.2014.00285

**Published:** 2014-12-23

**Authors:** Douglas K. Price

**Affiliations:** Molecular Pharmacology Section, Genitourinary Malignancies Branch, Center for Cancer Research, National Cancer Institute, National Institutes of HealthBethesda, MD, USA

**Keywords:** resistance, anti-cancer drugs, signaling pathways, cancer, transporters

While the majority of patients with cancer treated with chemotherapy initially respond to treatment, a sub-group of patients either do not respond or eventually stop responding to the therapy in a phenomenon known as tumor cell resistance. Evidence of many different mechanisms of resistance have been reported in the literature, and it remains a great challenge to delineate the pathways involved, and subsequently develop therapies to combat and overcome chemotherapy resistance. It is only in the understanding of the mechanisms of tumor cell resistance to cytotoxic drug-induced apoptosis at the molecular and biochemical levels that will allow the development of drugs that target the resistance factors.

Dr. Benjamin Bonavida is well-qualified to edit this book given his expertise as he has years of investigation, and well over 100 publications devoted to understanding tumor cell resistance. Dr. Bonavida has assembled a stellar cast of contributing authors that are clearly leaders in this field of study. The book, while quite comprehensive, is not designed to, nor does it attempt to cover the entire field of drug resistance. The focus of the book is to highlight specific mechanisms of cancer drug resistance and review or propose new treatments targeting each of these mechanisms. The contributing authors of the 12 chapters provide reviews of widely studied aspects of cancer drug resistance, present the latest findings, and offer speculative targets for novel drug development. Both tissue specific mechanisms/interventions and targets common to many cancers are included. The practice of single agent administration and the use of sensitizing drugs synergized with other therapies are also discussed. A brief overview of some of the chapters contained in the book follows.

The first chapter may be the most important one for readers that are not already familiar with cancer drug resistance. It gives an excellent overview of the phenomenon of multidrug resistance (MDR) by the overexpression of the ATP-binding Cassette (ABC) drug transporters. The chapter details the structure and function, substrate specificity, and the polymorphic effects on the three most important ABC genes. Small molecule targeted therapies and natural products as modulators (enhanced by well-referenced tables) are reviewed. This chapter is followed by a review that provides details on the role of pH changes that alter physical properties of the cell membrane and MDR functions. The remaining chapters each review important mechanisms and pathways that contribute to chemotherapy resistance and the treatment strategies to overcome this phenomenon.

An interesting chapter worth mentioning reviews what is currently understood about “collateral sensitivity,” or hypersensitivity to otherwise drug-resistant cells. This is a lesser-known multiple mechanism process in drug resistance, and a possible novel treatment strategy. The challenges of optimizing combination therapies, cross-resistance and hypersensitivity in multidrug-resistant cells are reviewed in detail. This chapter contains an especially helpful table describing collateral sensitivity in drug-resistant tumors broken down by currently used cell lines.

The Raf/MEK/ERK signaling pathways are often aberrantly activated in cancer and the description of the mechanisms of Raf inhibitor resistance and therapies to target this are discussed in two comprehensive chapters. The need for, and application of, combination therapies designed to co-target multiple aspects of signaling pathways is presented with an emphasis on personalized medicine. B-Raf as a target for anti-cancer agents and novel therapeutic strategies are reviewed.

In one of several chapters that focuses on a tissue specific cancer, the role of β 1 integrins and resistance to lung cancer is discussed. This chapter highlights the importance of the β 1 integrin-extracellular matrix signaling in tumorigenesis and subsequent drug resistance. The authors describe ongoing efforts to target the β 1 integrin as a potential breakthrough in the successful treatment of lung cancers. The aldo-keto reductase (AKR) genes as targets for chemoresistance in colon cancer are discussed in a separate chapter. Current literature focusing on expression profiles is reviewed and the accompanying figures of the important metabolic pathways are excellent.

Many anticancer agents induce Reactive Oxygen Species (ROS) that triggers apoptosis resulting in cancer cell death. A thorough chapter reviews the ROS molecular mechanism. The treatment approach to identify targets for elevating ROS levels as way of sensitizing drug-resistant cells is also discussed in detail. Well-written and referenced chapters that review the role of apoptosis, DNA repair and signal transduction pathways, TRAIL-apoptotic pathway, and cancer stem cells in resistance to cytotoxic drugs are also included in the book.

Overcoming cancer drug resistance has become a priority in the treatment of many types of cancer with drug-resistant cells becoming harder to kill even with the arrival of new cytotoxic drugs. The field is complicated due to the wide array of pathways and mechanisms involved. In conclusion, this well-organized book provides the reader with an excellent overview of the field of cancer drug resistance. The test is informative and comprehensive complemented by extensive references and helpful schema that make this book a valuable resource for students, those new to the field, and veteran investigators.

## Conflict of interest statement

The author declares that the research was conducted in the absence of any commercial or financial relationships that could be construed as a potential conflict of interest.

